# Role of the Built and Online Social Environments on Expression of Dining on Instagram

**DOI:** 10.3390/ijerph17030735

**Published:** 2020-01-23

**Authors:** Vishwali Mhasawade, Anas Elghafari, Dustin T. Duncan, Rumi Chunara

**Affiliations:** 1 Department of Computer Science & Engineering, Tandon School of Engineering, New York University, Brooklyn, NY 11201, USA; vishwalim@nyu.edu (V.M.); anas.elghafari@nyu.edu (A.E.); 2 Department of Epidemiology, Mailman School of Public Health, Columbia University, New York, NY 10032, USA; dd3018@cumc.columbia.edu; 3 Department of Biostatistics, College of Global Public Health, New York University, New York, NY 10003, USA

**Keywords:** Instagram, built environment, social media network, expression of dining

## Abstract

Online social communities are becoming windows for learning more about the health of populations, through information about our health-related behaviors and outcomes from daily life. At the same time, just as public health data and theory has shown that aspects of the built environment can affect our health-related behaviors and outcomes, it is also possible that online social environments (e.g., posts and other attributes of our online social networks) can also shape facets of our life. Given the important role of the online environment in public health research and implications, factors which contribute to the generation of such data must be well understood. Here we study the role of the built and online social environments in the expression of dining on Instagram in Abu Dhabi; a ubiquitous social media platform, city with a vibrant dining culture, and a topic (food posts) which has been studied in relation to public health outcomes. Our study uses available data on user Instagram profiles and their Instagram networks, as well as the local food environment measured through the dining types (e.g., casual dining restaurants, food court restaurants, lounges etc.) by neighborhood. We find evidence that factors of the online social environment (profiles that post about dining versus profiles that do not post about dining) have different influences on the relationship between a user’s built environment and the social dining expression, with effects also varying by dining types in the environment and time of day. We examine the mechanism of the relationships via moderation and mediation analyses. Overall, this study provides evidence that the interplay of online and built environments depend on attributes of said environments and can also vary by time of day. We discuss implications of this synergy for precisely-targeting public health interventions, as well as on using online data for public health research.

## 1. Introduction

Online social communities constitute a significant presence in our lives. Researchers and practitioners are using online data to illuminate many aspects of life including health, politics and culture, based on what people post [1,2,3,4,5]. For example, some studies have focused on recipe websites and shown how food names in online recipes can be a proxy for consumption and dietary patterns of individuals [6]. Given the growing literature that uses online data and social network behavior to understand health patterns and outcomes, there is an important missing piece. To better understand links between online behavior data and health outcomes, we need to understand the factors that contribute to online posting behavior for pertinent health-related topics. The importance of understanding what influences social media posting behavior has spurred a growing literature on this topic. For example, a recent study examined the relationship between online factors (such as post ephemerality, audience, etc.) and posting behavior on multiple social media sites and topics (including food) [7]. They found that posting behavior could be predicted by considering such characteristics of the online environment better than a random model. This is reasoned to be the case based on theory about how individuals perceive and value features of the online environment [7,8].

Better understanding of the process by which online data is generated will help public health researchers and practitioners improve analyses of population-level trends from social media data (by understanding what factors should be accounted for). As well, understanding what factors can influence online posts can help practitioners create more targeted interventions (by better understanding of health trends from online data and to create more focused interventions using information from both the offline and online worlds). Therefore, we explore online and offline environmental factors in relation to posting behavior and how they synergize in this paper. We focus on dining expression on social media due to active research on using big data sources for predicting and understanding behaviors and outcomes related to food consumption [9,10,11,12]. Given this body of work that is drawing conclusions based on what is posted online, there is an important need for further research to understand what influences people’s posts about food and dining.

Research in public health provides the theory to motivate variables we select in this study, that could potentially influence food posting behavior. First, food-related behaviors are known to be influenced by the built environment (e.g., by types of food available near us) [13]. Decades of public health research shows that what is around us in the built environment; for example, lack of sidewalks, long distances to schools, the need to cross busy streets, can influence our behaviors such as what we eat [13]. Moreover, recent changes in the nutrition environment, including greater reliance on convenience foods and fast foods are related to what people can access and consume [14]. Second, a major determinant of human eating behavior is social modeling, whereby people use others’ eating as a guide for what and how much to eat [15]. Literature indicates that such information can influence choice and quantity of food eaten, and as well could be used to promote healthy changes to dietary behavior [16]. Moreover, there is an increasing literature describing the online social environment (social reinforcement via likes and comments from the online social network) in regards to food consumption and health-related behaviors [17]. For example, linking facebook likes or foursquare checkins, and specifically Instagram food posts, to obesity at the population level (refs. [9,10]. Such influences have been hypothesized to occur by multiple rationales—-socially connected individuals share similar experiences, events, influences, and support which lead to similar behaviors, people choose to associate with others like them (homophily), and individuals exert influence on others [18,19]. It should be clarified that this study is not aimed at probing the reasons for these links within the online social environment, however given this research on the existence of social environment mechanisms, it is clear that we need to better understand how both built and social environments relate to online food posting behavior.

To-date, work that has examined relationships between online and offline phenomena has assessed mediating and moderating mechanisms [20]; motivating us to also assess these forms of mechanisms. Most closely related to the work here, Walker et al. performed a post-hoc mediation analyses finding that online physical appearance comparison and online fat talk could be mediating the association between Facebook intensity and disordered eating [21].

Given the existing research, we consider and measure one particular aspect of the built environment that has been well studied; the types of food options available in a user’s neighborhood [22,23]. For the social environment we consider the proportion of profiles in a user’s network that post about dining. A user’s dining posts are quantified by the proportion of posts that are dining related. Indeed, the drivers behind our online posting behavior may involve many offline and online variables, and the way these numerous variables may synergize is complex. Thus, choosing these initial variables and analyses represent a step forward to empirically illustrating these significant, yet not well understood relationships. Assuming posting disproportionately about unhealthy dining is undesirable, public health messaging and interventions could be targeted better based on knowing what factors from the built and social environment both matter. We thus study these factors through the lens of Instagram and in the city of Abu Dhabi. Abu Dhabi has both a vibrant social media and large culinary/restaurant-going culture [24,25]. In particular, it may be more common for people in Abu Dhabi to post photos of what they are doing (or, for example, eating), compared to photos of other people due to different cultural and legal privacy norms regarding expectations of privacy than in other parts of the world [26]. Moreover, this study may improve the presence of studies in places that are under-represented in the social media literature (a simple PubMed search on 29 December 2019 shows 99 results in IJERPH with the keyword Shanghai anywhere in the text (using the [tw] field in PubMed), 17 with Toronto, 28 with London, 40 with “New York City” and 3 with “Abu Dhabi”). Agnostic to location, the approach and findings in relation to the interplay of built and online environments should be considered as an exemplar study for food-related posting and other studies of content on social media in relation to topics of public health importance.

We address the following specific research questions:

- (i) What is the relationship between the built environment (measured through types of restaurants in neighborhood) and dining posting behavior by users who post in those neighborhoods? (ii) What is the relationship between the social environment (measured through proportion of a user’s social network in Abu Dhabi that posts about dining) and dining posting behavior by users in Abu Dhabi?
- Does the social environment moderate the relationship between the built environment and proportion of dining posts a user posts?
- Does the social environment mediate the relationship between the built environment and proportion of dining posts a user posts?

## 2. Methods

### 2.1. Data Collection

#### 2.1.1. Instagram Data

**Figure 1:**
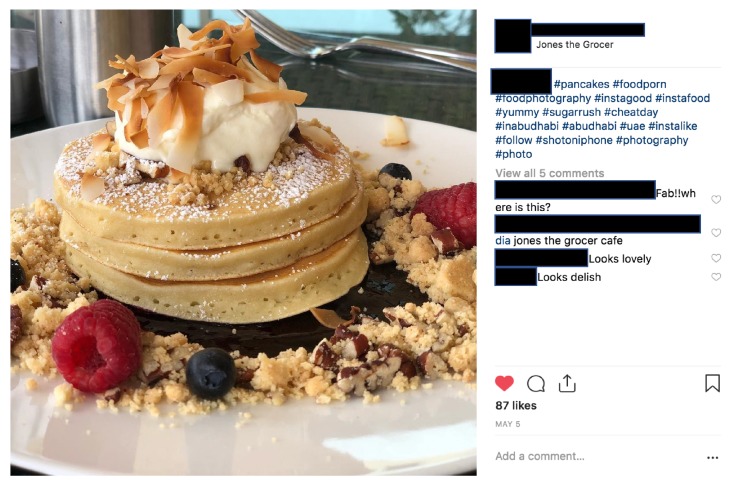
A sample dining-related Instagram post from Abu Dhabi.

We obtained Instagram data (posts with linked username, comments, captions and hashtags and likes) using an Application Programming Interface (API) provided by DialogFeed (https://www.dialogfeed.com/) from October 2017 to December 2017. After Instagram made changes to its API in 2016, only approved outside developers could directly access Instagram data. However, data is made available legally to select providers which mainly use the data for marketing purposes. Accordingly, we used the services of DialogFeed, an approved Instagram data feed provider. The data associated with each Instagram post available from Dialogfeed includes: the image, hashtags (for example; #photography, #uae), the number of likes, the date and time of the post and geo-location (if tagged by the user). Specific data collection steps, including to ensure posts distributed across Abu Dhabi were collected, and filtering posts to obtain those related to dining. We generated a 1302 word dictionary (including words in English, Hindi and Arabic) in order to filter posts related to dining. Comprehensive details about this dictionary and its development are in Appendix A. In sum, this resulted in 252,773 posts from users with public profiles, containing words that are present in the food-related dictionary (in any of the captions/hash tags/comments) with a geo-location within Abu Dhabi. Figure 1 shows a sample post. We select a sample of users for the study based on this data (explained in detail in Section 2.1.2). Given that it is not possible to obtain the profile of those in the network of a given user from DialogFeed, we had to manually gather the network information, explained further in Section 2.1.4.

#### 2.1.2. Sample Selection

**Figure A1:**
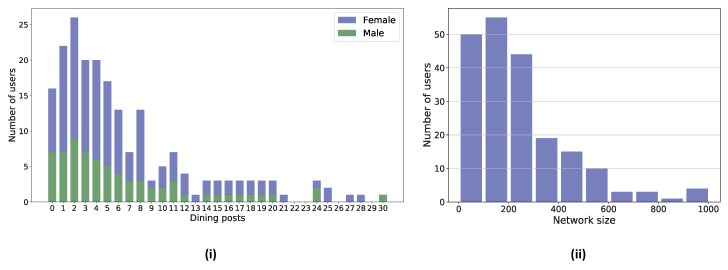
(**i**) Distribution of the dining posts (shown for both males and females) (**ii**) Network (number of profiles followed by a user) size distribution.

We identified specific users from Abu Dhabi for our study (and discluded people who are only visiting Abu Dhabi). Although tourists’ posts could be influenced by the environment they visit as well as the information in their social media network, as they are not residents, the influence of the local environment may not be consistent with the influence on residents, so we exclude them to focus the study on the locals.To accomplish this, our team manually scanned through the post and comment text as well as the images of each of the 96,050 Instagram users who generated a total 252,773 posts, out of which 894 Instagram users had posted about dining related content (even though they all were filtered by the same dining-related keywords and were captured by the DialogFeed API). We then filtered the users to determine if the user resides in Abu Dhabi by examining posts from their last 30 days. Based on the users’ posts we were able to assess who had simply traveled to the region recently, and excluded those users. We found that this time period and the type of posts were sufficient to classify someone as a resident. Accruing data from this time period and process identified a sample of 200 users residing in Abu Dhabi with dining related content. This sample size was deemed adequate given that we expect a small to medium indirect effect and partial mediation with a sufficient power (80%) [27], and was feasible to obtain within the data acquisition time period. As linked demographic information is not available via DialogFeed (or Instagram), our team also manually examined the profile (photo and handle) of each included user to identify their gender. We assigned each profile to a binary gender categorization. There were no cases where our team disagreed about the tourist versus local, or gender categorization. There are many unmeasured variables that can confound relationships, however this was the most simple potentially-related variable to infer, though we stress that it is an inference. Distribution of the number of dining posts by user in the resulting sample is illustrated in Figure A1i.

#### 2.1.3. User Dining Posts (Dependent Variables)

We then studied this sample of individuals residing in Abu Dhabi to get the following dining behavior related information: number of posts that have dining related content in their most recent 30 Instagram posts. We created a systematic protocol and criteria for counting something as a “dining” post, or not. This was achieved by examining not just the photo posted but the specific location, such as the specific restaurant (if included) as well as the hashtags, captions, and comments on the post. We found that the unclear posts were those about baked goods. This was discussed and resolved based by removing posts with hashtags that indicated baking (such as #homebaking, or #bakeshome). Other than baking posts, we did not find any discrepancies between our team members regarding the classification of posts as dining out, or not. We considered the latest 30 posts (from the date of analysis) in order to assess posts over a reasonable amount of time, while trading off the amount of time needed to examine the data. The minimum time span for 30 posts was 0 days (all 30 posts were made in a single day), maximum was 56 days and mean was 7 days. Rather than include proportion of Instagram posts that were dining related, we included the absolute number to represent the amount of dining posts as well as their general posting behavior, within a similar time period. Distribution of the number of dining posts by user in the resulting sample is illustrated in Appendix C. For added rigor in studying the varied influences on dining posts, we assessed the effects at different times of day. We consider three periods to capture different meal times; morning (posts made between 6:00 a.m.–12:00 p.m. local time), afternoon (12:00 p.m.–6:00 p.m.), and evening (posts made after 6:00 p.m.). Since we already have the time at which an individual posted (the time for each post) we determine the number of dining posts made by an individual in the morning (l${\mathrm{DP}}_{\mathrm{morning}}$), afternoon (${\mathrm{DP}}_{\mathrm{afternoon}})$ and evening (${\mathrm{DP}}_{\mathrm{evening}}$). The dependent variables considered are included in the Table 1 summary.

#### 2.1.4. Networks (Possible Mediator Variables)

The online social environment of an Instagram user consists of the Instagram profiles that a user follows. Before March 2018, the Instagram feed consisted only about the information that the profiles in a user’s network (profiles that the Instagram user chooses to follow) share (https://instagram-press.com/blog/2018/03/22/changes-to-improve-your-instagram-feed/). Later, the feed was personalized for each individual, but since our timeline does not extend beyond the date of this change, considering the Instagram profiles in a user’s network and their posts are relevant measures of the social environment for possible influence on a user’s posting behavior. To access and categorize the network profiles, given the large number of total profiles to consider, and given that it is not possible to obtain data from DialogFeed via the Instagram handle (only via keywords/locations), we had to manually query the Instagram search engine and automatically classify the location of profiles and posts of each user followed in order to obtain location information of the network profile and determine if it was Abu Dhabi based. Figure A1ii shows the distribution of the network sizes of the users. To study the network profiles (focusing only on the public profiles since the private profiles cannot be studied) we labelled each public profile that the user follows as either dining related (if the profile had any dining content) or not. We also delineated profiles as personal or business profiles. All four mediator variables are summarized in Table 1. Labels for the network profiles were assigned via an Amazon Mechanical Turk (AMT) task (details in Appendix B).

The possible mediator/moderator variables are thus—number of overall network profiles that post about dining, and the number of non-dining profiles. We use the absolute number of dining/non-dining profiles, instead of proportions in order to assess the possible effect of each individually.

#### 2.1.5. Mapping Posts and Users to Neighborhoods

**Figure 2:**
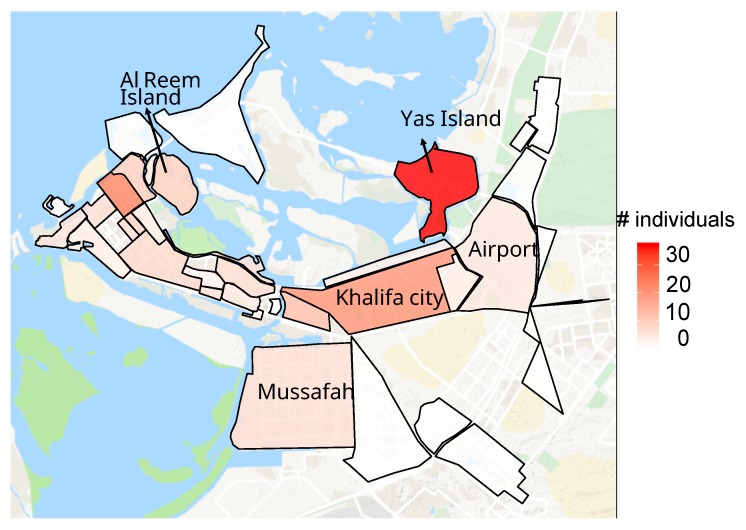
The distribution of users across Abu Dhabi neighborhoods (largest neighborhoods labelled).

As the number of each restaurant type is at the neighborhood level, we need to assign a neighborhood to each user; we select this based on the neighborhood most frequented by the user for dining purposes. It should be noted that this is not necessarily the neighborhood a user lives in. We divided the city into 23 neighborhoods based on categorization on Zomato and used the geo-location associated with each post to assign it to a neighborhood. Unlike other social media platforms like Twitter, each user’s profile does not have a general location associated with it on Instagram. However, the posts do have a geo-location and the posts are captured by DialogFeed based on this geo-location. Although a user might choose not to disclose the location of the post on the platform, Dialogfeed captures the posts based on the internal geo-location provided by Instagram which might not be made available by the user to their followers, but is still made available and accessible to DialogFeed; we therefore obtain the geo-location of along with the post. Using neighborhood boundaries from Google Maps, we generated a shape file to define these neighborhoods. This shape file was then used to map each user post to a neighborhood (this is necessary since we are interested in the neighborhood from which a post is made as opposed to the specific latitude/longitude of the post). Having the neighborhood of each user post enables us to then assign a unique neighborhood to each user. The neighborhood assigned is the one from which maximum number of posts are made by the user. The resulting neighborhood distribution of users is illustrated in Figure 2.

#### 2.1.6. Neighborhood Built Environment (Independent Variables)

**Figure 3:**
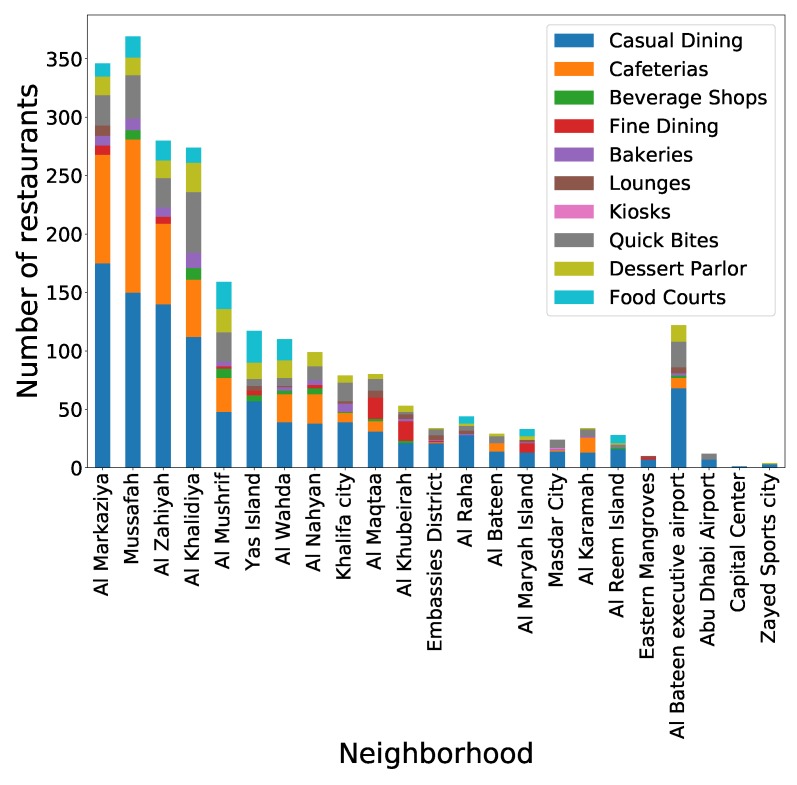
Distribution of restaurant types by neighborhood.

Potentially multiple aspects of the built environment can influence dining behavior and in turn dining posting behavior. A very well known influence on what a person eats is based on which restaurants are available in the immediate environment of a user [28]. Therefore based on this knowledge from public health research, we use data from Zomato (https://www.zomato.com) to determine the food environment (via type and number of restaurants) by neighborhood in Abu Dhabi. Zomato is a restaurant review website, similar to Yelp which is commonly used in other countries (a detailed list of the countries using Yelp can be found at: https://www.yelp.com/locations). The type of restaurants in several categories were aggregated by neighborhood. The types include: casual dining restaurants, cafeterias, fine dining restaurants, bakeries, lounges, kiosks, quick bite restaurants, dessert parlors and food court restaurants. These are summarized in Table 1. The distribution of restaurants by category and by neighborhood is also illustrated in Figure 3. Of note, we excluded the neighborhood Al Karamah as it consists largely of tourist destinations. Users assigned to this neighborhood were mapped instead to the second-most common neighborhood in their posts. We included these different categories as it may be intuited that each restaurant type may play a different role in its influence on dining posting. Therefore each of the restaurant types are independent variables of interest.

#### 2.1.7. Ethics and Privacy

Given the sensitive nature of social media research, we are committed to securing privacy and minimizing any possible risks. We have not disclosed any individual-level content in this publication. We discussed the tasks to our institution’s IRB for their suggestions on best practices for working with the data (though informed the study is not under their purview for evaluation as human subjects research, based on public nature of the social media data).

### 2.2. Analysis Approach

To test research question one part i), we first test the relationship between the built environment via neighborhood restaurant categories (the independent variable, IV), and a user’s dining posting behavior on Instagram (the dependent variable, DV) in the absence of the network features. We do so using a regression analysis, the results of which are presented in Table 2. Next, we test the relationship between the social environment (profiles in an individual’s social environment on Instagram (*M* variable) and a user’s dining posting behavior on Instagram (the dependent variable, DV) also using a regression analysis; detailed results are presented in Table 3.

#### 2.2.1. Moderation Analysis

To explore the effect of *M* as a moderator on the relationship between IV and DV, we examine the statistical significance of *M* as a covariate in the relationship between the IV and DV. A moderator is generally understood to be a relationship which affects the strength of the relationship between the IV and DV. With the variables considered here, one may interpret this as if the association between restaurants in one’s neighborhood and a user’s online dining posts will be moderated by dining profiles in the user’s social network such that this association between presence of restaurants in one’s neighborhood and online dining posts will be more pronounced among those who have more dining profiles in their social network, compared to those who have less dining profiles in their network. As we have four possible mediator variables, we perform one regression for each *M* variable. Table 4 represents the mean and standard deviation across the four regressions, for each independent variable. After confirming the statistical significance of *M* variables in a regression (moderator) analysis, given previous research that has found mediation relationships between online and offline phenomena, we also if the relationship between the IV on DV operates via *M*; that is, if *M* may *explain* the partial or full relationship between IV and DV (research question three). In our case one may interpret this as if the association between restaurants in one’s neighborhood and their dining posts is partially (or fully) explained by what a user sees in their online social network.

#### 2.2.2. Mediation Analysis

**Figure 4:**
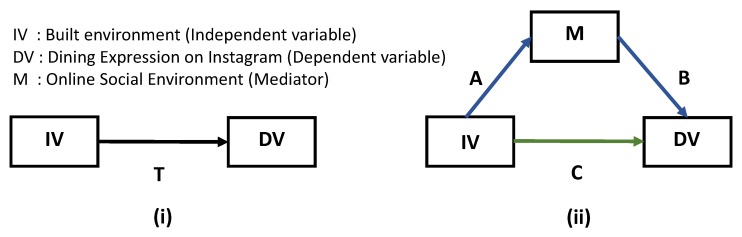
Diagram illustrating the mediation model: (**i**) denotes the total effect of the independent variable on the dependent variable (**ii**) denotes the indirect effect of the independent variable on the dependent variable through the mediator variable (paths A, B)) and the direct effect (path C).

The most widely used method to assess mediation is the causal step approach outlined in the classic work of Baron & Kenny [29]. Four steps are involved in establishing mediation. To begin, the direct relationship between IV (in our case we actually consider multiple treatments, via the restaurant categories: bakeries, lounges etc.) by neighborhood and the DV (Instagram dining posts by users in those neighborhoods) is assessed using a multi-level regression model (*step 1*: IV→DV). Multi-level models are used here to account for the hierarchical nature of the data (individuals, neighborhoods) since multiple users are assigned to the same neighborhoods. Next, the association between each of the significant independent variables (restaurant features) (p<0.05) in step 1 and the potential mediator variables were tested with multi-level regression (*step 2*: IV→*M*). A third model tested the relationship between the network features (mediator: *M*) and outcome variable (user dining posts) (*step 3*: *M*→DV). If a feature had a significant association with the number of dining profiles in the network from 1 and 3, then they were both entered into a final regression model to test their joint association with user dining posts (*step 4*: IV→*M*→DV, IV→*M*). If the restaurant features (IV) became insignificant and the mediator (features of the network) remained significant, the relationship between built environment and online dining posts was determined to be fully mediated. If both the restaurant measures and the network features remained significant, the relationship was determined to be partially mediated. If the mediator (network measures) did not affect the relationship between restaurant features and individual posting (restaurant features remained significant) and the mediator itself becomes insignificant, no mediation occurs. The process is represented in Figure 4ii. In sum, the relationships can be described via a “total effect” (between IV and DV, in the absence of *M*), “direct effect” (effect of IV directly on DV in the presence of mediator *M*) and “indirect effect” (effect of the independent variable through the mediator, IV→*M*→DV). The total effect represents the regression coefficients of the built-environment types from *step 1* (IV→DV). These estimates are reported in Table 5. The regression coefficients of the same built-environment types from *step 4* (IV→*M*→DV, IV→DV) constitute the direct effect. Table 5 also presents the direct effect of IV on DV in presence of *M*. Confidence intervals are obtained by bootstrapping. The indirect effect (effect of IV on DV via *M*) is calculated by subtracting the direct effect from the total effect. In all models we used a Poisson regression based on the identified distributions (Figure A1i,ii and Figure 3). As secondary analyses, we perform mediation analyses with different DVs, besides the total number of dining posts made by an individual; dining posts made in the morning, dining posts made in the afternoon, dining posts made in the evening. This was studied to understand effect of mediation at different times of day, when different meals are commonly eaten. All variables were standardized and all statistical analyses were conducted using R version 3.4.3.

## 3. Results

### 3.1. Descriptive Analysis of Posts

We qualitatively examined the distribution of hashtags and content of the posts by user, as well as likes on posts by each user to understand if there were any patterns regarding types of food by neighborhood, or other measures of social desirability/perception that could potentially inform further variables for the study. The top 10 hashtags were: *#abudhabifood*, *#mac&cheese*, *#streetfood*, *#meat*, *#buffet*, *#pizza*, *#diner*, *#salad*, *#foodphotography*, and *#desserts*. We found a similar distribution of these top hashtags across the neighborhoods. This consistency is notable; the expression via hashtags (and potentially types of foods that are pictured) does not vary across neighborhoods, even though the general frequency of dining expression does.

### 3.2. Moderation Analysis Results

Moderation analysis results are presented in Table 4. First, the total adjusted $R^{2}$ of the model increases after inclusion of the moderator variable; meaning more of the variance in the IV is explained if the moderator variable is included. Further, we note that the interaction terms (IV * *M*) being statistically significant has a positive effect on the DV. We have multiple interaction terms and each of these are significant, indicating that interaction of the built and social environments help better explain the variance in IV. We further notice that the regression coefficients of #Category: Delivery and #Food Courts change signs after introducing the moderator and the interaction term, supporting that the moderator (dining posting in the social network) has an impact on the relationship between the IV and DV. The number of non-dining profiles was negatively associated with each independent variable and was not found to be significant. The number of personal or business profiles was not significant in the model, hence those coefficients are not reported, and further analyses aggregate over all of these types of network profiles (personal and business).

### 3.3. Mediation Analysis Results

Without considering any mediating variables, we found that bakeries and lounges (lounges are places people generally hang out at night in Abu Dhabi) were positively associated with a higher proportion of dining posts in a user’s timeline (Table 5, total effects, Appendix D full regression results). Food court restaurants, casual dining restaurants (more expensive restaurants than food court restaurants) were negatively associated with Instagram dining posts. In other words, as the number of bakeries and lounges increases in a neighborhood, we are more likely to see dining out photos on Instagram by users located in those neighborhoods.

Examining the direct, indirect and total effects (Table 5) shows, first, that we did see at least small-medium effect sizes (based on the thresholds in [27]). Further, for the number of casual dining, bakeries and lounges in a neighborhood, the total and indirect effects have the same direction. Therefore, the effect of having more of these types of restaurants in a user’s neighborhood is the same as the effect of these types of restaurants propagated through their social network. On the other hand, for the number of food court restaurants, the total and indirect effects are negative, but the direct effect is positive. For these types of restaurants for which the direction (positive or negative sign) of the effect is inconsistent, we can understand that while the direct effect may be in one direction (say, more food court restaurants in a neighborhood is associated with an increased number of dining posts in a user’s profile; perhaps users may be likely to post about eating at food court restaurants), the indirect effect is in the opposite direction, meaning these types of restaurants are related to a (in this case) decrease in the number of dining posts in a user’s profile. Therefore even though the direct effect of having more restaurants of this type in the neighborhood is associated with an increase in the number of dining posts, an increase in food court restaurants is not something that propagates its effect through the network (suggesting users are not inspired to post about these places based on their online social network). The total effect for the number of food court restaurants is thus negative even though the direct effect is positive.

### 3.4. Mediation Results Across Different Times of the Day

**Figure 5:**
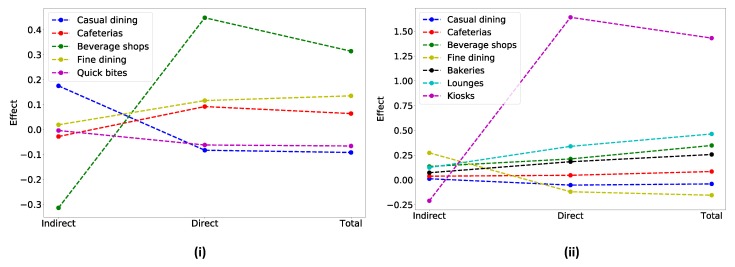
Indirect, direct and total effect of significant restaurant categories in (**i**) afternoon and (**ii**) evening (no significant effects in morning).

Based on mediation analyses with the same restaurant types (IV) and the mediator variables (# dining profiles in the user network) based on user posts made during different time periods, we found that there is no significant (p<0.05) mediation effect in the morning. Comparing the mediation effects in the afternoon with evening we find that a higher number (7) of restaurant categories (casual dining, cafeterias, beverage shops, fine dining, bakeries, lounges and kiosks) have significant effect in the evening while the restaurant categories (casual dining, cafeterias, beverage shops, fine dining, quick bites) having a significant effect in the afternoon is considerably lower (5). It also should be noted that the magnitude of the mediation effect is significantly more in the evening than afternoon. The afternoon and evening mediation effects for the different restaurant categories are illustrated in Figure 5i,ii respectively.

We found that casual dining has the highest mediation effect (indirect effect) in the afternoon while fine dining has the highest mediation effect in the evening. This can be intuited, since fine dining restaurants cater to specialized menus and are frequented by users in the evening. Causal dining restaurants on the other hand do not necessarily always provide sit ins and have general menus offering meal options for lunch. We also found that kiosks had the most negative indirect effects in the evening. This could be due to the casual nature of these types of restaurants, and thus these effects are not mediated through the online environment. In general, we find that effect trends in the afternoon by restaurant are different from those in the evening.

## 4. Conclusions

### 4.1. Interpretation of Findings

The broad results of this study, include that first, there is a relationship between attributes of the built environment and Instagram posting behavior. Further, that those relationships vary by attributes (types of built environment locations, times of day) and can be mediated by the social environment. In sum, the online social environment can act as a mediator of the relationship between the built environment and expression of dining on Instagram. Specifically, we found that the mediation relationship identified here is not consistent by type of built environment variable (e.g., type of restaurant) nor is it constant throughout the day. Notably, we found no significant mediation effect in the morning; while there was a significant mediation effect in both afternoon and evening. We further found that the mediation effect in the evening is higher than afternoon and also the effect is significant for more restaurant categories in the evening than afternoon. Differences in social pressures on eating behaviors at evening time have also been described in the eating behavior literature which shows that people’s pre-existing personal preferences might reduce social modeling at times when people have clear eating routines or scripts regarding regular meals such as breakfast and lunch [15]. Notably, food courts were the restaurant category that were not significantly mediated at either afternoon or evening. Given that food courts are a mass establishment, with multiple food vendors (and focus on fast food establishments), social desirability or possible mechanisms of the social environment effect may be decreased. While we can use social media to quantify important neighborhood-level characteristics that would be otherwise difficult to measure [30], such studies tend to focus on data from specific locations and generalizability is still an important question. It is not possible to assume that specific results regarding relationship between specific dining categories and online posting behavior would be the same in other places. We picked Abu Dhabi for good reason; it has varied neighborhoods, an active dining culture, active social media environment, and is understudied in social media research. The findings regarding role of both the built and online environments in posting behavior on social media, as well as approach used here (mediation analysis) can act as exemplars for further social media research.

In terms of specific findings by restaurant-type in Abu Dhabi, we found that the number of bakeries and lounges in a user’s neighborhood were positively associated with an increase in the number of dining posts in the user’s Instagram timeline (positive direct effect: Table 5). We also found a positive indirect effect in the mediation analysis for these restaurant types, therefore we can infer that this effect is positively mediated by the individual’s Instagram network. This would be interpreted such that users would be more likely to post about these types of locations in their environment, and this effect may be in part because of what kinds of posts are in the social network. There could be multiple mechanisms for these findings. Potentially, for these types of locales, people are positively inspired to post about based on content in their online social networks. There also could be mechanisms related to the communal aspect of such places, and/or the types of food. Results may also inform further study of the mechanism for the effect for different restaurant categories at different times of day (e.g., quick bites did not have a significant social mediation effect in the evening, though they did in the afternoon).

On the other hand, casual dining restaurant types (which are a more expensive type of place than fast food restaurants); showed negative direct and total effects suggesting people are less likely to frequent and post about these types of places, and this trend may also be in part due to what is posted in the online social network (positive indirect effect). Finally, while the total effect for food court restaurants was negative, just having these types of restaurants in a user’s neighborhood had a positive direct effect though the indirect effect was negative (Table 5). This suggests that these are potentially directly associated with more dining posts, but as above, less socially desirable types of restaurants (the sign of the effect flips when considering direct effect compared to total effect).

### 4.2. Limitations

There are important limitations to note from this work that can help drive future studies. As discussed above, the specific findings regarding restaurant types are in relation to Instagram use in Abu Dhabi (a significant majority of the Abu Dhabi population uses social media and of those over half use Instagram [24], which is popularly used for sharing dining posts [12]). Overall, our offline and online interactions are complex. Indeed, geo-tagged social media posts cannot capture the full extent of places we visit, and other variables measuring impression management, emotional management or self-presentation are also important factors influencing posting behavior [31,32]. This profusity of variables is echoed by low $R^{2}$ of the regression models in the moderator analysis. Although the mean adjusted $R^{2}$ was ∼17% when including the the number of dining profiles mediator variable this is approximately an eight-times increase over the $R^{2}$ for the model without this variable. These values are both still low, indicating that there are many more variables that will explain variance in the outcome, however it also shows the importance of the social environment variable. Overall in terms of variables, we selected specific variables grounded in the public health and social media literature, to exemplify a specific relationship and overall, and findings from this first study on the physical and online food environments, do show that there is an interaction between considered variables and give an idea of how that relationship can be be modelled. Naturally, socio-economic and demographic variables, which are very important in public health studies should be incorporated, as available, to gain a richer understanding of any effects. As well, information that may delineate the types of people who share geo-located posts on Instagram, with others, would also be valuable. In sum, this work should activate further research identifying more of the complexities by considering both environments, as well as understanding influences on different social media platforms and populations in different places.

### 4.3. Future Work

Future work can incorporate image processing to identify objects in the Instagram posts. Further data collection could also be done to add a temporal dimension to the posts from users and their network to establish temporal precedence in the mediation, and also consider post-specific built-environment mediation effects, not just those for a user’s home built environment. The analysis here still draws important conclusions based on text in the posts, and follows much work in social media analysis that has focused on this text [12,17,33]. Regarding the analysis, regardless of the identified mediational path for dining posting behaviors, because these are cross-sectional data, future longitudinal studies, including annotation of photo locations over time (instead of considering the home location of each user) would require more complex mediation models, but be necessary in order to make causal inferences. This may also increase the amount of data required over time. There are also several important assumptions for tests of mediation. We briefly describe these here, though they are articulated in more detail in other sources [29]. Assumptions include no mis-specification due to unmeasured variables that cause relation in the mediation analysis (we used all possible variables though there is always the effect of unmeasured variables that are important), and no misspecification due to imperfect measurement (in our case that could be incompleteness of the restaurant list or our samples did not represent the overall posting behavior of users and their networks well) [34]. These analytic assumptions are known to be difficult or impossible to test; accordingly the general approach is to build on information from prior research, including experimental studies and theory, as we have done here, to strengthen the conclusion that a mediation relation exists.

### 4.4. Importance and Utility of Findings

Social media is playing an increasing role in our lives; the way that people come together on these systems to share and ingest information, especially related to food posting, has been illustrated in recent social media research [2,11]. Research here advances this work; beyond characterizing what is posted online and how that relates to what is posted in one’s online network, we go further to understand the relationship between the built and social networks, in food posting on social media.

Uncovering the relationship between the physical/built environment, social environment and our food posting behaviors can be helpful to inform the design of health-promoting technologies. For example, in areas with particularly poor physical food-environments (high concentration of fast food restaurants, for example), if the information on posts from the social network was consented to be shared, public health agencies, companies, or social media platform designs may invest in pro-active online messaging, as also suggested through the eating behavior literature [16]. Moreover, given the social environment and mediation results presented here (research questions two and three), messaging could be targeted with precision to those more vulnerable to posting (and assumed consuming of) unhealthy foods based on information exposed to from the online social network beyond just those who may live or be near restaurants with unhealthy food. Given the multi-level nature of factors in public health, we add that this study can motivate and contribute to research on how attributes of a restaurant beyond the type of food are relevant to eating behaviors; which is to say that the types of organizations as well as the food they sell can be intertwined in their effect on our behaviors and health [35]. These findings can also help researchers understand influences on posting behavior, so that empirical factors that contribute to posts can be accounted for when drawing conclusions from population-scale social media studies (e.g., we should account for offline environmental factors when we conclude that county *X* has less healthy posts than county *Y*).

## Figures and Tables

**Table 1:** Summary of variables tested: independent variables (IV), mediator variable (*M*) and dependent variable (DV).

Feature (Number of)	Feature Description	Mean	Range (Min, Max)
IV:	
Casual dining	Eateries with general menu	35.32	(0, 187)
Cafeterias	Counters/stalls serving food	20.08	(0, 164)
Beverage shops	Shops serving juices/beverages	1.67	(0, 10))
Fine dining	Formal settings catering selected menu	2.06	(0, 18)
Quick bites	Informal setting with specific menu	9.17	(0, 52)
Bakeries	Shops serving baked items	2.15	(0, 13)
Lounges	Formal setting serving food, drinks	1.15	(0, 9)
Kiosks	Small shops/carts serving specific menu	0.15	(0, 3)
Food Courts	Indoor setting inside malls	4.86	(0, 27)
*M*:	
Dining profiles	Network profiles that post about dining	59.60	(0, 381)
Non-dining profiles	Network profiles that do not post about dining	183.91	(0,966)
Personal profiles	Personal profiles in the users’ network	163.32	(0, 915)
Business profiles	Business profiles in the users’ network	79.89	(0, 538)
Gender	Gender of the user; female (F) represented as 1	0.66	F: 120, M: 80
DV:	
$DP_{\mathrm{total}}$	Total dining posts made by the user in 30 days	6.51	(0, 30)
$DP_{\mathrm{morning}}$	Dining posts made during morning	0.86	(0, 10)
$DP_{\mathrm{afternoon}}$	Dining posts made during afternoon	2.21	(0, 18)
$DP_{\mathrm{evening}}$	Dining posts made during evening	3.62	(0, 24)

**Table 2:** Regression results for the relationship between the built environment (IV) and user’s dining posting behavior on Instagram (DV): IV→DV with DV being the total number of dining posts made by an individual.

Feature (Number of)	β	std.err	*p*
*Intercept*	0.1240	0.0021	0.0410 *
IV:	
Casual dining	−0.4389	0.0012	<0.0001 ***
Cafeterias	0.0646	0.0263	0.1132
Beverage shops	0.3140	0.1280	0.1424
Fine dining	−0.1162	0.0352	0.9801
Quick bites	−0.6641	0.0176	0.8710
Bakeries	1.4124	0.0012	0.0328 *
Lounges	0.6158	0.0310	0.0190 *
Food Courts	−0.3475	0.0002	<0.0001 ***

*** p<0.001, ** p<0.01, * p<0.05, .p<0.1.

**Table 3:** Regression results for the relationship between the social environment (*M*) and user’s dining posting behavior on Instagram (DV): M→DV with DV being the total number of dining posts made by an individual.

Feature (Number of)	β	std.err	*p*
*Intercept*	0.1093	0.0010	0.0320 *
M:	
Dining profiles	1.5042	0.0001	0.0019 ***
Non dining profiles	0.0001	0.0901	0.0910 .
Personal profiles	0.0210	0.1210	0.0600 .
Business profiles	0.0092	0.0222	0.1008

*** p<0.001, ** p<0.01, * p<0.05, .p<0.1.

**Table 4:** Regression coefficients from Moderator analysis (interaction term = IV * M), *Dining profiles* is the only moderator variable presented here since it was the only moderator variable that improved the $R^{2}$ value of the model.

	Without Moderator	With Moderator
Feature (Number of)	(Mean Adjusted R-Squared:	(Mean Adjusted R-Squared:
	0.0238)	0.1687)
*Intercept*	0.1341 *	0.1091 *
IV:
Casual dining	−0.438 ***	−0.343 **
Bakeries	1.412 *	0.049 *
Lounges	0.615 *	0.078 *
Food Courts	−0.347 **	0.025 *
*Moderator (M):*
Dining profiles	-	0.170 *
*Interaction terms:*
Causal dining * M	-	0.011 *
Bakeries * M	-	0.011 *
Lounges * M	-	0.010 *
Food Courts * M	-	0.012 *

*** p<0.001, ** p<0.01, * p<0.05, .p<0.1.

**Table 5:** Mediation results: total, direct and indirect effects (number of dining profiles as the mediator variable) with total dining posts $DP_{\mathrm{total}}$ as the dependent variable. Regression coefficient with 95% CI. Only the significant (p<0.05) independent variables (IV) are reported. Total effect represents the regression coefficients of IV without considering the mediator variable (*step 1:*IV→DV), Direct effect represents the regression coefficients of IV in *step 4*: IV→M→DV,IV→DV. The indirect effect is the difference between the these two values.

IV	Total Effect	Direct Effect	Indirect Effect
(Number of)			
Casual dining	−0.44 (−1.96, 1.08)	−1.41 (−2.12, −0.70)	0.97
Bakeries	1.41 ( 0.12, 2.71)	0.27 (−0.21, 0.75)	1.14
Lounges	0.62 ( 0.02, 1.21)	0.18 (−0.07, 0.44)	0.44
Food courts	−0.35 (−0.56,−0.14)	0.40 ( 0.22, 0.57)	−0.74

## Appendix A. Filtering Instagram Data

Data access via DialogFeed is limited via certain endpoints (by specifying the hashtags associated with posts and/or geo-locations of the posts), thus we collected data as follows. The location from which posts were made (posts are linked with exact latitude/longitude) was restricted to the area within the geographical boundaries of Abu Dhabi. Twenty locations along with an appropriately sized radius (5 km) were used to ensure that posts made from every part of Abu Dhabi were captured. In order to filter by content, following current research that utilize hashtags to understand Instagram content [12,33,36,37], we filtered posts that involved dining related content in the post’s linked hashtags and comments, based on keywords in a food dictionary we developed (future work could involve multi-modal analyses of both the image and text content). We created the dictionary by scraping and aggregating all food words from menus of the restaurants in Abu Dhabi on Zomato. We used the restaurant menus to capture names of cultural dishes as well as general food items available in the restaurants in Abu Dhabi. For example; the hashtags for the example post of pancakes in Figure 1 include the word: *pancakes*. There are a total of 1302 words in the dictionary (including words in english, hindi and arabic). The focus was to identify posts specifically in relation to dining out and not related to cooking activities or grocery shopping of food items. Although posts related to activities like cooking are indicative of the dining behavior of an individual; they are not directly reflective of the influence of the built environment on expression of experiences of dining posts on social media. Therefore, posts showing consumption of meals at home, cooking, raw ingredients, or packaged snacks were not considered dining related as they are not relevant to the dining experience being considered. Also, we excluded terms that would be solely indicative of beverages, coffee, tea, alcohol drinks, since the factors that relate to posting meals may very well differ from those relating to posting coffee or drinks, etc. Finally, we also observed common hashtags and words used on dining-related Instagram posts that are not related to food, so added in these. Examples of included words (in english) are: *brownies, casseroles, dining-out, eatout, fastfood, gelatos, hotdogs, icecream, jelly, kebabs, lobsters, meatless, nougat, organic, pancakes, quesadillas, rasberries, sandwiches, tempura, upma, veggies, waffle, yogurt, and zucchini.*

## Appendix C. Network Variables

The task of annotating the profiles in the Instagram network of an individual consisted of first categorizing a profile as personal or business (described as cafes, brands, etc.). The second task involved determining if there is any dining related content in the first 30 profile posts (this is conjunction to identifying the dining-related content posted by an Instagram user as mentioned in Section 2.1.3). To ensure the accuracy of the task each network profile is categorized by two independent workers on AMT. The inter-rater agreement was 70%. In case of a disagreement between the two, we sought a third label from AMT and obtained the majority vote for the annotation. Our team manually also examined 20% randomly sampled labels provided by AMT turkers to check for the accuracy. This in conjunction with a high inter-rater agreement convinced us to use the labels obtained from AMT. We extracted the location associated with each of the first 12 posts of each profile in the network of each user to determine if the profile was Abu Dhabi based (12 posts was picked to balance time, given there were 39,747 total network profiles, and to give a long enough time span, on average, by which to determine the overall location of the network profile). It is important to assess the network posts from Abu Dhabi profiles, as these may be influenced by the built environment as well (Figure 5ii) and the effect must be decoupled. If the majority of the posts of a single network profile are made in Abu Dhabi, then we consider that profile to be physically in Abu Dhabi. For those profiles in Abu Dhabi, we assign the profile to the neighborhood of the user it is associated with. At the end, each profile in the network has a neighborhood and a profile type (dining or not dining, personal or business) associated with it.

## Appendix D. Detailed Regression Results

Here expand on the details of the mediation analysis for complete clarity on each of the mediation analyses. For the primary and secondary analyses, we perform four mediation analyses in total; the dependent variables being number of dining posts in the morning ($DP_{morning}$) presented in Table A1, number of dining posts in the afternoon ($DP_{afternoon}$) presented in Table A2, number of dining posts in the evening ($DP_{evening}$) presented in Table A3 and the total number of dining posts ($DP_{total}$) presented in Table A4.

**Table A1:** Summary of regression results of mediation analysis: independent variables (IV), mediator variable (*M*) and dependent variable (DV) being dining posts made during the morning ($DV_{morning}$) between 6:00 a.m.–12:00 p.m. The results for IV are presented with *Dining profiles* as the mediator variable.

Feature (Number of)	β	std.err	*p*
*Intercept*	0.5509	0.1356	0.0081 **
IV:	
Casual dining	−0.1823	0.0981	0.0642 .
Cafeterias	1.8492	0.4309	0.0852 .
Beverage shops	−0.0128	0.9191	0.0567 .
Fine dining	0.8913	0.0109	0.0632 .
Quick bites	0.1268	0.5171	0.1200
Bakeries	−1.0831	0.5432	0.0924 .
Lounges	0.3421	0.0004	0.0761 .
Kiosks	0.0942	0.4005	0.5412
Food courts	0.2556	0.1067	0.0616 .
*M*:	
Dining profiles	0.9481	0.4321	0.1002
Non dining profiles	0.0021	0.0042	0.2021
Personal profiles	0.0231	0.0428	0.0600 .
Business profiles	0.0421	0.0231	0.2009
Gender	0.0031	0.0012	0.1200

*** p<0.001, ** p<0.01, * p<0.05, .p<0.1.

**Table A2:** Summary of regression results of mediation analysis: independent variables (IV), mediator variable (*M*) and dependent variable (DV) being dining posts made during the afternoon ($DV_{afternoon}$) between 12:00 p.m. and 6:00 p.m. The results for IV are presented with *Dining profiles* as the mediator variable.

Feature (Number of)	β	std.err	*p*
*Intercept*	−0.5454	0.2002	0.0064 **
IV:	
Casual dining	−0.0838	0.0194	0.0017 **
Cafeterias	0.0992	0.0204	0.0001 ***
Beverage shops	0.4188	0.1054	0.0023 **
Fine dining	−0.1358	0.0347	0.0001 ***
Quick bites	−0.0623	0.0183	0.0006 ***
Bakeries	0.0089	0.4562	0.4367
Lounges	0.0010	0.5732	0.6752
Kiosks	−15.4985	0.1431	0.9900
Food courts	−7.8610	0.1293	0.7861
*M*:	
Dining profiles	0.3413	0.0010	0.0011 **
Non dining profiles	−0.0021	0.0005	0.3210
Personal profiles	0.0098	0.0023	0.1287
Business profiles	0.0023	0.0391	0.6542
Gender	0.0053	0.0013	0.0891 .

*** p<0.001, ** p<0.01, * p<0.05, .p<0.1.

**Table A3:** Summary of regression results of mediation analysis: independent variables (IV), mediator variable (*M*) and dependent variable (DV) being dining posts made during the evening ($DV_{evening}$) after 6:00 p.m. The results for IV are presented with *Dining profiles* as the mediator variable.

Feature (Number of)	β	std.err	*p*
*Intercept*	0.4736	0.1111	0.0002 ***
IV:	
Casual dining	−0.5231	0.0161	0.0011 **
Cafeterias	0.4772	0.0143	0.0008 ***
Beverage shops	1.0128	0.0491	0.0001 ***
Fine dining	−0.1199	0.0273	0.0001 ***
Quick bites	−0.0468	0.0171	0.0621 .
Bakeries	0.1841	0.0452	0.0042 **
Lounges	0.3391	0.0684	0.0001 ***
Kiosks	1.6142	0.4275	0.0001 ***
Food courts	−3.4536	0.0067	0.9749
*M*:	
Dining profiles	2.0085	0.0004	0.0001 ***
Non dining profiles	0.0002	0.2901	0.3400
Personal profiles	0.0010	0.7810	0.2297
Business profiles	0.0302	0.0832	0.6000
Gender	0.0001	0.3405	0.1000

*** p<0.001, ** p<0.01, * p<0.05, .p<0.1.

**Table A4:** Summary of regression results of mediation analysis: independent variables (IV), mediator variable (*M*) and dependent variable (DV) being total dining posts ($DV_{total}$). The results for IV are presented with *Dining profiles* as the mediator variable.

Feature (Number of)	β	std.err	*p*
*Intercept*	0.1736	0.1011	0.0001 ***
IV:	
Casual dining	−1.4101	0.0112	0.0002 ***
Cafeterias	0.9390	0.0043	0.0700 .
Beverage shops	0.0438	0.0021	0.1001
Fine dining	−0.1199	0.0219	0.3001
Quick bites	−0.1408	0.0198	0.6002
Bakeries	0.2684	0.0422	0.0029 **
Lounges	0.1802	0.0124	0.0007 ***
Kiosks	0.1142	0.0325	0.0891 .
Food courts	0.3997	0.1027	0.0042 **
*M*:	
Dining profiles	1.5042	0.0001	0.0019 **
Non dining profiles	0.0001	0.0901	0.0910.
Personal profiles	0.0210	0.1210	0.0600 .
Business profiles	0.0092	0.0222	0.1008
Gender	0.0081	0.1205	0.0901 .

*** p<0.001, ** p<0.01, * p<0.05, .p<0.1.
